# Virtual Screening for Potential Allosteric Inhibitors of Cyclin-Dependent Kinase 2 from Traditional Chinese Medicine

**DOI:** 10.3390/molecules21091259

**Published:** 2016-09-21

**Authors:** Fang Lu, Ganggang Luo, Liansheng Qiao, Ludi Jiang, Gongyu Li, Yanling Zhang

**Affiliations:** Beijing Key Laboratory of TCM Foundation and New Drug Research, School of Chinese Material Medica, Beijing University of Chinese Medicine, Beijing 100102, China; lufang1017@163.com (F.L.); 17801080765@163.com (G.L.); b20100222012@163.com (L.Q.); jiangludi_52@163.com (L.J.); lidoc2727@163.com (G.L.)

**Keywords:** CDK2, allosteric inhibitors, pharmacophore, molecular docking, TCM

## Abstract

Cyclin-dependent kinase 2 (CDK2), a member of Cyclin-dependent kinases (CDKs), plays an important role in cell division and DNA replication. It is regarded as a desired target to treat cancer and tumor by interrupting aberrant cell proliferation. Compared to lower subtype selectivity of CDK2 ATP-competitive inhibitors, CDK2 allosteric inhibitor with higher subtype selectivity has been used to treat CDK2-related diseases. Recently, the first crystal structure of CDK2 with allosteric inhibitor has been reported, which provides new opportunities to design pure allosteric inhibitors of CDK2. The binding site of the ATP-competition inhibitors and the allosteric inhibitors are partially overlapped in space position, so the same compound might interact with the two binding sites. Thus a novel screening strategy was essential for the discovery of pure CDK2 allosteric inhibitors. In this study, pharmacophore and molecular docking were used to screen potential CDK2 allosteric inhibitors and ATP-competition inhibitors from Traditional Chinese Medicine (TCM). In the docking result of the allosteric site, the compounds which can act with the CDK2 ATP site were discarded, and the remaining compounds were regarded as the potential pure allosteric inhibitors. Among the results, prostaglandin E1 and nordihydroguaiaretic acid (NDGA) were available and their growth inhibitory effect on human HepG2 cell lines was determined by MTT assay. The two compounds could substantially inhibit the growth of HepG2 cell lines with an estimated IC_50_ of 41.223 μmol/L and 45.646 μmol/L. This study provides virtual screening strategy of allosteric compounds and a reliable method to discover potential pure CDK2 allosteric inhibitors from TCM. Prostaglandin E1 and NDGA could be regarded as promising candidates for CDK2 allosteric inhibitors.

## 1. Introduction

Cyclin-dependent kinases (CDKs) belong to the serine/threonine kinase family, which are key regulatory enzymes in cell division [1]. Cell division is one of the cell-cycle processes and acts as an essential role in the production of new cells. The cell-cycle process including four phases, named G1 (gap 1), S (synthesis), G2 (gap 2), and M (mitotic) phase. The transition from one phase to another is regulated by series of cellular proteins, especially CDKs and cyclins. Owing to the critical role of CDKs in the control of cell division, deregulation of CDKs can lead to abnormal processes and numerous human diseases, most notably cancer and tumors. Therefore, suppression of CDK activity serves as an ideal therapeutic strategy for cancer and tumor by interrupting aberrant cell proliferation [2].

Cyclin-dependent kinase 2 (CDK2) is an important member of CDKs, which complex with cyclinA/E, mainly controlling the G1-to-S phase checkpoint and DNA replication [3]. Hence, given its pivotal role in cell cycle regulation, CDK2 has been actively regarded as a promising drug target for anticancer therapies. The CDK2 inhibitors can arrest and recover control of the cell-cycle in aberrantly dividing cells [4,5]. There are two types of CDK2 inhibitors. One is the classical CDK2 inhibitors, namely ATP-competitive inhibitors, which directly bind to the ATP site or extend from the ATP site into the adjacent allosteric site; the other one is the allosteric CDK2 inhibitors, which bind to the allosteric site, away from the ATP site. The classical CDK2 inhibitors such as flavopirido had been a failure in clinical trials due to the low subtype selectivity, which lead to unwanted off-target interactions and undesired toxicity [6]. On the contrary, an allosteric inhibitor can supply higher selectivity and prolong the time of drug action [7]. So, designing allosteric inhibitors of CDK2 is imminent.

The crystal structure of CDK2 complexed with pure allosteric inhibitor, 8-anilino-1-naphtalene sulfonate (ANS), has been reported [8]. In the crystal structure, two ANS molecules interact with allosteric site and locate near to each other in the vicinity of the C-helix, which is away from the ATP site. ANS is the first known extrinsic compound that interacts with the allosteric binding site with proven high affinity. Binding of compounds with allosteric nature is often accompanied with significant conformation changes in CDK2 [6]. A large conformational change appeared in the C-helix when bound with ANS, which suggested that this site has potential to disrupt the interaction of CDK2 and cyclin A [8]. It had been reported that ANS could inhibit the catalytic activity of the phosphorylated CDK2-cyclin A2, suggesting that the presence of the allosteric inhibitors can obstruct the formation of the complex between CDK2 and cyclins [8]. This is the first time the allosteric mechanism of CDK2 has been clearly resolved, which provides a new opportunity to design pure CDK2 allosteric inhibitors. Hence, it is highly desirable to design compounds with a higher affinity to interact with this newly discovered allosteric pocket.

Traditional Chinese medicine (TCM) has been widely used to relieve and treat various cancers and tumors with good effects and lower side-effects [9,10]. A number of ingredients of TCM have been reported as having efficacy by interacting with the allosteric site [11]. For example, magnolol and honokiol can treat anxiety and convulsions by targeting the allosteric site in GABAA [12]. In recent years, molecular simulation technologies, such as molecular docking, pharmacophore model, and molecular dynamics were used to discover new drugs from TCM and elucidate the mechanism at the molecular level [13]. For instance, Tang et al. discovered several CDK2 ATP-competitive inhibitors from TCM by using molecular docking and molecular dynamics [9]. However, the discovery of pure CDK2 allosteric inhibitors from TCM has not been carried out.

**Figure 1:**
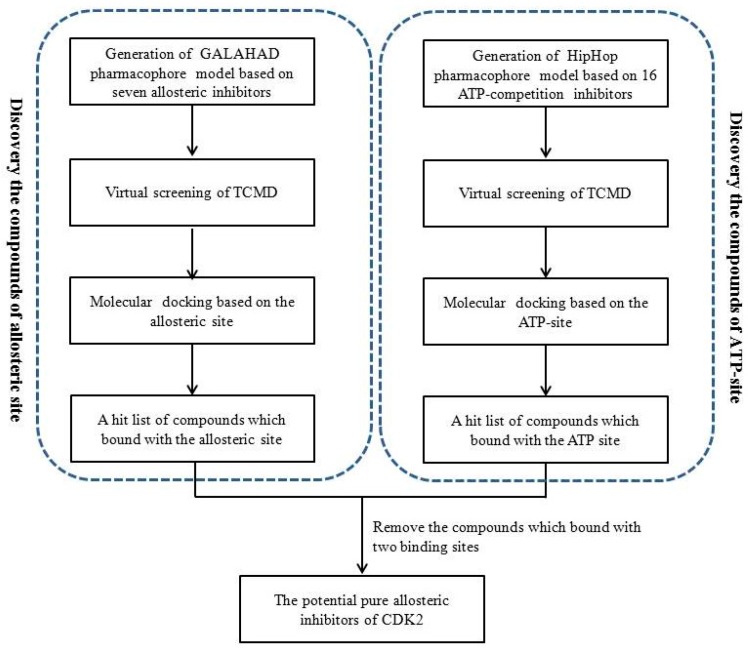
Virtual screening strategy of this study.

The purpose of this study was to screen potential pure allosteric inhibitors of CDK2 from TCM by using a series of molecular simulation methods and MTT assay would be used to test the growth inhibitory effect of the screened compounds. The same compound might interact with the two binding sites, as the binding sites of two types of CDK2 inhibitor are overlapped in space position. Thus a new screening strategy of the pure CDK2 allosteric inhibitors was presented (Figure 1). In this study, Ligand-based pharmacophore models, including GALAHAD models were constructed based on the known allosteric inhibitors and the common feature pharmacophore models (HipHop) were generated based on the active CDK2 ATP-competition inhibitors, respectively. The optimal models were used as queries to screen potential CDK2 allosteric inhibitors and ATP-competition inhibitors from TCM. Meanwhile, the structure-based method, molecular docking, was also utilized to refine the above screening results. The screened compounds which can interact with both two binding site (ATP site and allosteric site) were discarded, and the potential pure allosteric compounds were chosen from the remaining compounds. Finally, two compounds with higher query fit value (QFIT) and higher molecular docking score were selected as the most promising CDK2 allosteric inhibitors. Given the CDK2 allosteric inhibitors with anti-cancer effect, the first known extrinsic allosteric inhibitor, ANS, was acted as a positive control in the MTT assay. The growth inhibitory effect of the most promising compounds and ANS was determined on human HepG2 cell lines which suggested the screened compounds should interact with the allosteric pocket of CDK2 to play the anti-cancer effect.

## 2. Results

### 2.1. GALAHAD Pharmacophore Hypothese Construction

**Figure 2:**
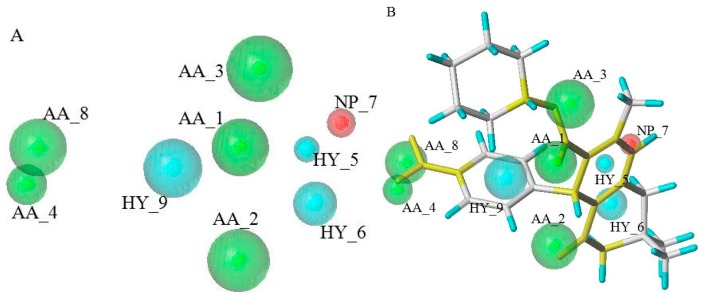
The optimal GALAHAD pharmacophore model of CDK2 allosteric inhibitors (**A**) and the allosteric compound BAS00380830 mapped with the optimal GALAHAD pharmacophore model (**B**). AA means hydrogen bond acceptors; HY means hydrophobic features; NP means positive nitrogen.

Twenty GALAHAD models were generated based on seven CDK2 allosteric inhibitors. The top eight pharmacophore models with higher “N_hits” were shown in Table 1. Based on the criteria, the values of “N_hits” should be six or seven. In this case, the value of “N_hits” was seven, which suggested that each model mapped all compounds in the training set. The PARETO rank was “0”, which indicated that none of the models was superior to others. From Table 1, according to the criterion, MODEL_002 and MODEL_003 with high energy which distributed in the different orders of magnitude were excluded [14]. MODEL_004 which had the lowest specificity was also eliminated. Among the remaining models, the energy values were distributed in the same order of magnitude. In order to increase the selectivity and specificity of the screening results, MODEL_007 which had the highest specificity and features was selected as the best model to screen the CDK2 allosteric inhibitors from TCMD. MODEL_007 which shown in Figure 2A had nine features, including five hydrogen bond acceptors (AA_1, AA_2, AA_3, AA_4, AA_8), three hydrophobic features (HY_5, HY_6, HY_9), and one positive nitrogen (NP_7). Seven CDK2 allosteric inhibitors in the training set were mapped with the optimal pharmacophore model, which showed all these nine features in the MODEL_007 was important to the compounds with allosteric nature. Figure 2B showed the allosteric compound BAS00380830 mapped with MODEL_007.

### 2.2. HipHop Pharmacophore Hypotheses Studies

#### 2.2.1. HipHop Pharmacophore Hypotheses Generation

Ten HipHop pharmacophore models were generated based on 16 active CDK2 ATP-competitive inhibitors and the results were detailed in Table 2. Four types of features were in the resulted pharmacophore models, containing A, H, D, and R. Each pharmacophore model had one A, one R, and one H, which suggested that these three features were important to active CDK2 ATP-competitive inhibitors. In Table 2, the Rank scores extended from 130.168 to 133.141 among the generated pharmacophore models, which suggested that they would be well-mapped and the models were reliable.

#### 2.2.2. HipHop Pharmacophore Model Validation and Optimization

**Figure 3:**
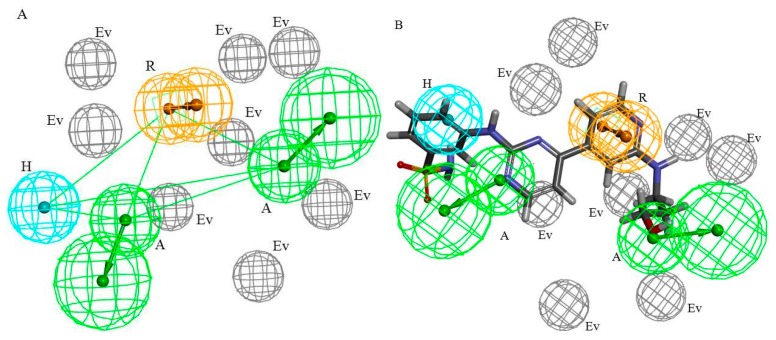
The best HipHop pharmacophore model of CDK2 ATP-competitive inhibitors (**A**) and the compound BDBM50394183 mapped with model Hypo1-1 (**B**).

A test set including 23 active CDK2 ATP-competitive inhibitors and 69 inactive compounds was used to validate the generated pharmacophore models. The evaluation indicators *HRA*, *IEI*, and *CAI* were used to choose the best pharmacophore model among 10 models. The validation results of 10 pharmacophore models were showed in Table 2. From Table 2, based on the Rank score, Hypo1 with the highest Rank score was selected to be optimized in next step. During the optimization procedure, three Excluded Volumes (Evs) were added to Hypo1. In order to reduce the hit rate of inactive compounds, the radius of eight Evs was increased by adjusting the tolerance of Evs. Then, the optimized model, Hypo1-1, was validated by the test set and training set. The compounds in the training set were all mapped with model Hypo1-1 successfully. Among the training set, BDBM50394183 was mapped with the pharmacophore model Hypo1-1, which showed in the Figure 3B. The evaluation indicators *HRA*, *IEI*, and *CAI* of model Hypo1-1 were 86.96%, 2.67, and 2.32, respectively. The value of *IEI* and *CAI* were increased which indicated the model Hypo1-1 had a greater ability than Hypo1 to distinguish active compounds from inactive compounds. Finally, the best pharmacophore model, Hypo1-1 (Figure 3A)—containing two A, one H, one R, and eight Evs—was served as a query to screen the TCMD.

### 2.3. Database Searching

The GALAHAD pharmacophore MODEL_007 of CDK2 allosteric inhibitors and the HipHop pharmacophore model Hypo1-1 of CDK2 ATP-competitive inhibitors served as queries to screen TCMD. The QFIT in GALAHAD and the Fit value in HipHop were calculated for ranking the matching rate of each hit, and a high QFIT value or Fit value indicated that the compound can map well with the pharmacophore models [15]. However, it was not a sufficient strategy to choose all these compounds for the next study. Then, the hit compounds were subjected to drug-likeness prediction by Lipinski’s rule of five (≥4). In this case, 2477 compounds were retained by the GALAHAD MODEL_007 and a list of 487 compounds was obtained by the HipHop pharmacophore model Hypo1-1. Finally, the two lists of compounds with drug-like properties were docked into the active sites, including the allosteric binding site and ATP binding site corresponding by using a molecular docking algorithm in DS (Discovery Studio 4.0).

### 2.4. Molecular Docking Studies

#### 2.4.1. Molecular Docking Studies of Allosteric Site

The allosteric binding pocket was created with a radius of 10.16 Å around the ANS1 and ANS2 presented in the crystal structure. Two docking algorithms, LibDock and CDOCKER, were used to evaluate their applicability for the docking study. The smaller RMSD value of the better of the docking algorithms [16], CDOCKER, which obtained the smaller RMSD value of 0.77 Å (<2.00 Å), was selected for the docking study. In addition, the −CDOCKER_ENERGY of ANS1 was 7.061, which was set as threshold to screen potential CDK2 allosteric inhibitors. The ANS1 formed the hydrogen bond interactions with LYS33, ASP145, and PHE146, and formed hydrophobic interactions with TRY15, PHE80, LEU55, LYS56, LEU66, LEU78, LEU55, and ILE52. Moreover, ANS1 also formed electrostatic interaction with LYS33. It was worth mentioning that these interactions were verified by the reported literatures [8,17]. Seven CDK2 allosteric inhibitors in the training set were docked into the allosteric binding pocket successfully, which elucidated the allosteric binding pocket was reasonable. All the CDK2 allosteric inhibitors can form hydrophobic interactions with LYS56, ILE52, LEU76, and LEU37. Hence, the binding mode of the screened potential CDK2 allosteric inhibitors should be similar to the above-mentioned binding mode of the CDK2 allosteric inhibitors.

#### 2.4.2. Molecular Docking Studies of ATP Site

**Figure 4:**
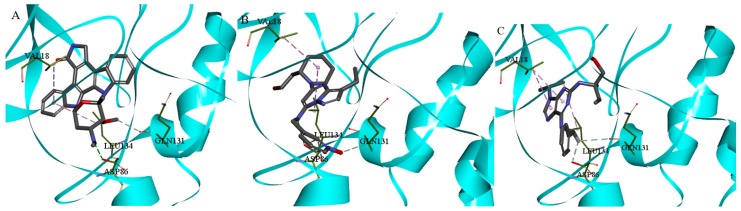
Docking results between Staurosporine (**A**); Dinaciclib (**B**); and Roscovitine (**C**) and CDK2 ATP binding site.

The conformation of CDK2, which can be combined with ATP-competitive inhibitor, was obtained by molecular dynamic. The RMSD value was 1.87 between the reported CDK2-staurosporine structure and the modified conformation and the TM-score was 0.931, which suggested that the two conformations were in approximately the same fold [18]. The active pocket of ATP site was defined with a radius of 15.00 Å by the residues Lys33, Asp86, Leu83, Asn132, Lys89, and Asp145 [6]. Fifteen CDK2 ATP-competitive inhibitors were all successfully docked into the ATP active binding pocket by CDOCKER, which was then chosen for the further docking study to analyze the binding mode and key residues of the CDK2 ATP-competitive inhibitors. For example, three CDK2 ATP-competitive inhibitors, Staurosporine, Dinaciclib, and Roscovitine formed hydrogen bond interactions with ASP86 and GLN131 and formed hydrophobic interactions with VAL18 and LEU134. The docking results between the three CDK2 ATP-competitive inhibitors and CDK2 ATP binding site were showed in Figure 4. The binding mode of CDK2 ATP-competitive inhibitors was consistent with the reported literature [6]. It indicated that the modulated conformation and the active pocket of ATP site were reasonable.

### 2.5. Selection of the CDK2 Allosteric Inhibitors

A list of compounds was retained by using the optimal GALAHAD model (MODEL_007) of CDK2 allosteric inhibitors and then they were docked into the allosteric active pocket. Similarly, a list of compounds was obtained by using the best HipHop model (Hypo1-1) of CDK2 ATP-competitive inhibitors, and then they were docked into the ATP active pocket. The compounds which interacted with the two binding sites were eliminated to improve the reliability of the results and recognize the pure allosteric inhibitors. Finally, 667 compounds, which only interacted with the allosteric binding site, were the pure CDK2 allosteric inhibitors.

Taking QFIT value and docking score into consideration, prostaglandin E1 and nordihydroguaiaretic acid (NDGA) might be the most promising pure CDK2 allosteric inhibitors. Prostaglandin E1 scored a −CDOCKER_ENERGY of 27.295 and a QFIT value of 26.840. According to the pharmacophore MODEL_007 mapping result, six features were mapped well with prostaglandin E1. Prostaglandin E1 interacted with amino acid LYS33, ASP145, and PHE146 forming hydrogen bonds and also formed hydrophobic interactions with residues LEU76 and HIS71. Moreover, the oxygen atom which mapped with AA_2 also formed hydrogen bonds with LYS33 and ASP145, respectively. It suggested that both the results of the GALAHAD pharmacophore model and molecular docking were consistent.

**Figure 5:**
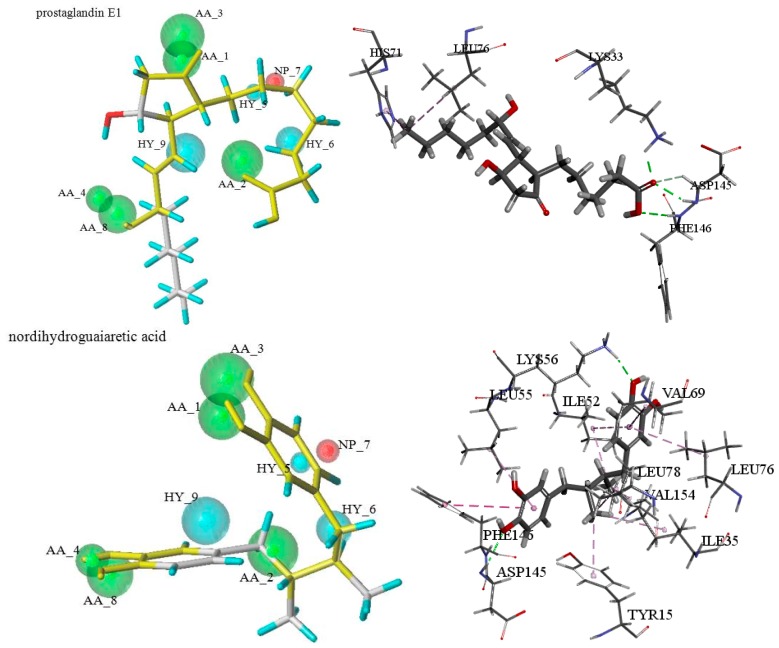
Pharmacophore mapping results and molecular docking results of prostaglandin E1 and nordihydroguaiaretic acid.

NDGA mapped six features with pharmacophore MODEL_007 and the QFIT value was 12.920. This potential compound got a −CDOCKER_ENERGY of 42.660, and formed hydrogen bond interactions with amino acids LYS56 and ASP145, also formed hydrophobic interactions with LEU55, LEU78, LEU76, and ILE52 and so on. Additionally, a benzene ring that matched with HY_5 also formed a hydrophobic interaction with residue LEU55 and PHE146. An oxygen atom mapped with AA_3 which also formed hydrogen bonds with ASP145. It can be seen that the results of the GALAHAD pharmacophore model were almost consistent with the molecular docking. Furthermore, the binding modes of these two potential pure CDK2 allosteric inhibitors were alike with the modes of the initial compound in the crystal structure. The two hits mapped with the corresponding pharmacophore models and the interactions with 3PXF were shown in Figure 5 and the growth inhibitory effect of them on human HepG2 cell lines would be tested by MTT assay.

### 2.6. Cell Proliferation Assay

**Figure 6:**
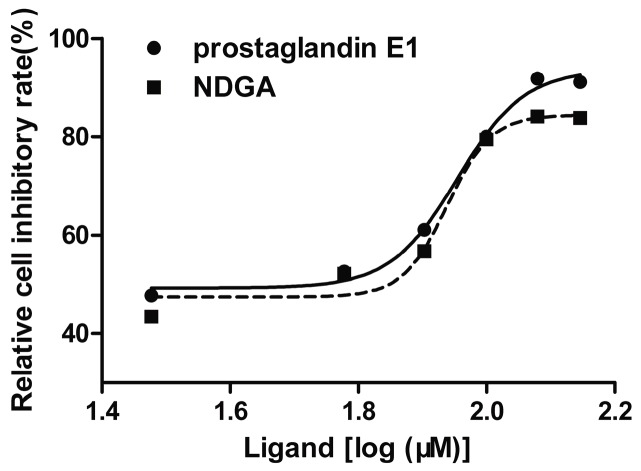
Effect of prostaglandin E1 and NDGA on cell proliferation in cultured HepG2 cells.

To test the proliferation effect of prostaglandin E1 and NDGA on cancer cell lines, HepG2 cell lines were treated with various doses of the two compounds and the positive compounds ANS, respectively, for 24 h and cell viability was examined by the MTT assay. Though ANS may not have reacted well selectively to many proteins in the cell, it is a certain pure CDK2 allosteric inhibitor. We selected ANS as the positive control, on account of the lack of marketed drugs using CDK2 allosteric inhibitor. The results indicated that ANS displayed a dose-dependent inhibition (0, 10, 30, 50, 80, 100, 120, and 150 μmol/L) with the estimated IC_50_ being 25.888 μmol/L. The tested IC_50_ of prostaglandin E1 was 41.223 μmol/L and NDGA was 45.646 μmol/L, respectively, at different concentrations of 0, 30, 60, 80, 100, 120, and 140 μmol/L (Figure 6). It was indicated that the two promising pure CDK2 allosteric compounds showed moderate-to-good activity with IC_50_ values in the μM range and indirectly suggested that the screened compounds, through interaction with the allosteric site of CDK2, play the role of inhibiting of cell growth.

## 3. Materials and Methods

### 3.1. Pharmacophore Modeling Studies

Seven CDK2 allosteric inhibitors were obtained from literature [17] to construct a pharmacophore model. GALAHAD is a powerful tool for pharmacophore modeling, especially suitable for the active compounds which share some structural commonalities [19]. As the seven CDK2 allosteric inhibitors with similar structure and IC_50_, the GALAHAD module in SYBYL-X 2.1 was used to construct pharmacophore model.

Thirty-nine CDK2 ATP-competitive inhibitors were obtained by entering “human CDK2 inhibitors” as a search term in the Binding Database (http://www.bindingdb.org/). The active CDK2 ATP-competitive compounds had the following characteristics: (1) The number of the active compounds was vast; (2) The structures were diverse; (3) The activity values of the compounds spanned a large range. The HipHop module within DS is appropriate for the active compounds with structural diversity to generate pharmacophore models [20]. Hence, based on the characteristics of the active CDK2 ATP-competitive compounds, the HipHop module was selected to generate CDK2 ATP-competitive inhibitors pharmacophore models.

#### 3.1.1. GALAHAD Pharmacophore Hypotheses Construction for the CDK2 Allosteric Inhibitors

**Figure 7:**
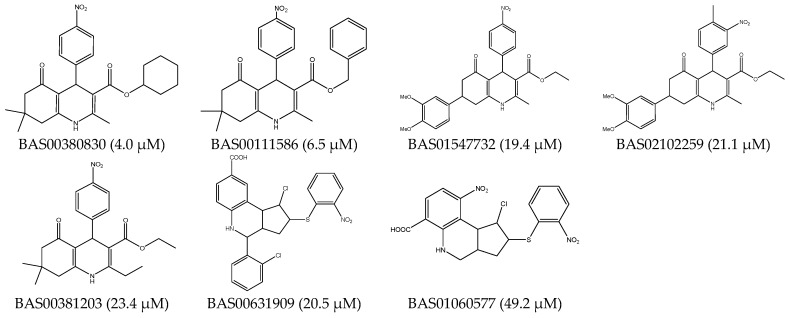
Chemical structures and IC_50_ of the compounds in the training set for the construction of GALAHAD pharmacophore model.

Seven active CDK2 allosteric inhibitors were defined as a training set to construct the GALAHAD model and all the compounds in the training set were minimized by the standard Tripos force field and the conjugate gradient method was utilized to carry out the energy minimizations with 1000 iterations. Hydrogen atoms were added to them and partial atomic charges were also added by Gasteiger-Hückel method. Chemical structures of the training set for the construction of the GALAHAD pharmacophore model were shown in Figure 7.

During GALAHAD operations, seven active compounds were aligned flexibly to each other in internal coordinate space. A genetic algorithm (GA) [21] was used to distinguish a list of conformations of compounds which had minimized energy and maximized pharmaco-steric similarity. Then, the produced conformers were considered as rigid bodies and aligned in Cartesian space. Linear assignment methodology and geometric heuristics were used to recognize the best feature correspondences between conformers [17].

The constructed models were validated based on three standards to choose the optimal model: (1) The number of “N_hits” and the number of active compounds should be approximately equal; (2) To have reasonable energy, the model ought to be within the same order of magnitude when compared with the others; (3) The model should have the maximum pharmacological features to increase the selectivity and specificity of the model [14].

#### 3.1.2. HipHop Pharmacophore Hypotheses Generation for CDK2 ATP-Competitive Inhibitors

##### HipHop Pharmacophore Hypotheses Generation

**Figure 8:**
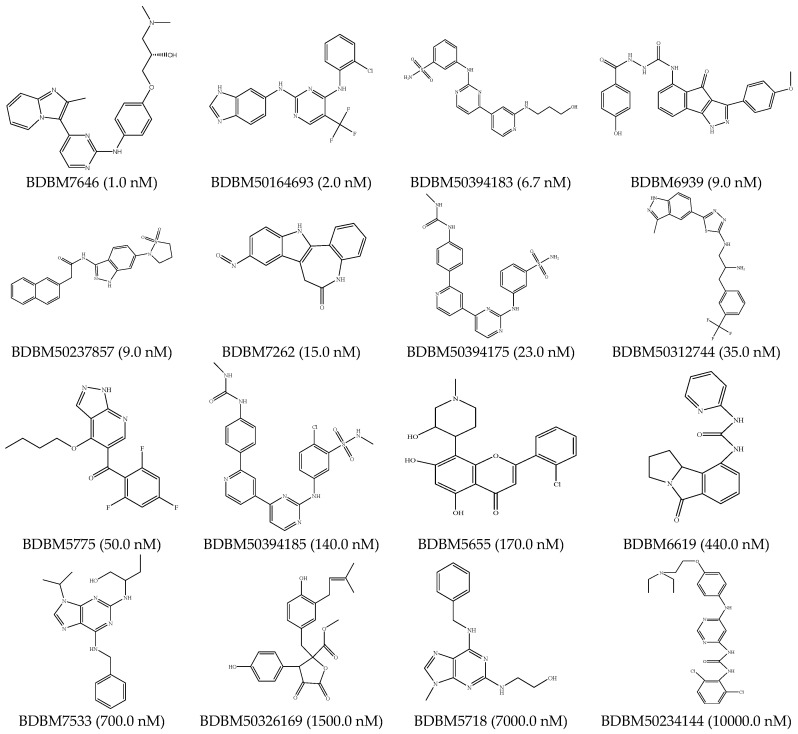
Chemical information of the training set for the HipHop pharmacophore model generation.

Among the 39 active CDK2 ATP-competitive inhibitors, 16 compounds which can represent the structural diversity were chosen as the training set for pharmacophore model generation. The IC_50_ of the compounds in the training set were across a range from 1.0 nmol/L to 10,000 nmol/L. Chemical information of the training set was shown in Figure 8. The 3D pharmacophore hypothesis for CDK2 ATP-competitive inhibitors was constructed by HipHop. Besides, the remaining 23 CDK2 ATP-competitive inhibitors were defined as active compounds in the test set. Another 69 compounds chosen randomly from the Binding Database were regarded as inactive compounds in the test set.

All compounds were first generated in 3D structure and fully minimized in CHARMm force, filed with MMFF94 partial charge. In order to make the conformation of compounds more close to the active conformation, multiple conformations of every compound were created by the BEST method and the maximum number of the generated compound conformations was set to 255. The relative energy threshold was 20 kcal/mol. Four pharmacological features, containing hydrogen bond acceptor (A), hydrogen bond donor (D), hydrophobic (H), and ring aromatic (R), were identified by the Feature Mapping study based on structures in the training set, and were chosen for pharmacophore generation.

During the generation process of HipHop pharmacophore model, the Principal value means the level of molecular activity and the corresponding MaxOmitFeat value indicates how many features were allowed to miss for each molecule. Hence, for BDBM5718 and BDBM50234144 with the lowest activity values, the Principal values were set to 0 and the corresponding MaxOmitFeat values were set to 2. BDBM50326169, BDBM7533, BDBM6619, and BDBM5655 had slightly higher activity values than the two lowest, both the Principal values and the corresponding MaxOmitFeat values were set to 1. For the remaining compounds, the Principal values were set to 2 and the corresponding MaxOmitFeat values were set to 0. In addition, the parameters of Feature Misses and Complete Misses were set to 1 and 0, separately. The Maximum Ev value was set to 5, and the other parameters were set to default values.

##### HipHop Pharmacophore Hypotheses Validation and Optimization

The test set including 23 CDK2 ATP-competitive inhibitors and 69 inactive compounds was used to evaluate all the HipHop pharmacophore models [15,22]. The evaluation indicators are presented as follows: *HRA*, *IEI*, and *CAI*. *HRA* means the ability of the pharmacophore model to recognize active compounds in the test set. The higher value of *HRA* shows the stronger ability of a pharmacophore model to recognize active compounds. *IEI* indicates the ability to distinguish active compounds from inactive compounds. With a high value of *IEI*, a hypothesis shows a strong ability to distinguish active compounds from inactive compounds. Then, *CAI* is the comprehensive appraisal index, which considers *HRA* and *IEI* at the same time, to evaluate the model comprehensively. Taking every factor into consideration, a superior pharmacophore model was selected for further study.

In order to improve the ability of the HipHop pharmacophore model to identify CDK2 ATP-competitive inhibitors, an optimization procedure was carried out. There are three parameters that can be manually adjusted, namely Tolerance (T), Distance Tolerance (DT), and Excluded Volume (Ev). T shows the radius of the sphere of each pharmacological feature. The size of the sphere represents the accuracy of the location for the pharmacodynamics characteristics; the smaller radius indicates that the size is more important for the activity. DT is the distance between each of the pharmacological features and the default value is ±1 Å. Ev is a crucial form of spatial constraint, which can characterize the spatial location of the ligands. Ev can be added by using Steric Refinement with Excluded volume protocol in DS based on the structural difference between the high active compounds and the low active compounds in the training set. By adding the Ev, the space constraint of the pharmacophore and the space complementary of the interaction between ligands and receptor can be strengthened.

### 3.2. Database Search

The best optimal GALAHAD and HipHop models would be used to screen potential compounds from Traditional Chinese Medicine Database (TCMD, version 2009) [23], which includes 23,303 compounds from 6735 medicinal plants. The Flexible search method was used to operate the screening processes. The hit compounds were filtered by “Lipinski’s rule of five” to retain the drug-like compounds, which must meet four rules, containing Hydrogen Bond Donors ≤ 5, Hydrogen Bond Acceptors ≤ 10, Molecular Weight ≤ 500, and AlogP ≤ 5 [24]. Finally, two lists of compounds with drug-like properties were obtained. One was the potential CDK2 allosteric inhibitors and the other one was the potential CDK2 ATP-competitive inhibitors. Then, the two lists would be further analyzed by molecular docking study, respectively.

### 3.3. Docking Studies

#### 3.3.1. Docking Studies of Allosteric Site

3PXF, a crystal structure of CDK2 in the RCSB Protein Data Bank (http://www.rcsb.org/pdb/home/home.do), was used as a reference model for molecular docking. The Prepare Protein protocol was carried out to add hydrogen, build loops, and assign suitable protonation states for 3PXF [25]. The same two CDK2 allosteric inhibitors, ANS, binding with the protein structure, one was named ANS1 (residue 305 in 3PXF) and the other one was ANS2 (residue 304 in 3PXF). The binding pocket of CDK2 allosteric inhibitor was created around the ANS1 and ANS2 using the Define and Edit Binding Site tools.

Two docking protocols, LibDock and CDOCKER, were used to test their applicability for the docking study of 3PXF. Compared with ANS2, ANS1 had a relatively good combination with the protein [17]. So ANS1 was defined as a reference to calculate RMSD value. After being extracted from the binding site, ANS1 and ANS2 were Re-docked into 3PXF. In general, the RMSD with less than 2.00 Å indicated that the docking algorithm would reproduce the experimentally observed binding mode for CDK2 allosteric inhibitors [20]. By comparing the RMSD value between experimental and computed structures of ANS1, the docking protocol that obtained the smallest RMSD was chosen for further utilizing.

In order to discuss the binding mode and the key residues of CDK2 allosteric inhibitors, seven CDK2 allosteric inhibitors from the training set of GALAHAD pharmacophore model were docked into the binding site. Then the poses and the key residues of the active compounds were analyzed. The list of potential CDK2 allosteric inhibitors, which were obtained by the GALAHAD model based virtual screening, were also docked to 3PXF using the selected docking protocol. Finally, the compounds, which had a docking score higher than the score of ANS1 and interaction with crucial residues which are similar to the CDK2 allosteric inhibitors, would be selected as potential CDK2 allosteric inhibitors.

#### 3.3.2. Docking Studies of ATP Site

##### Modulate the Protein Conformation to the ATP-Competitive Inhibitor Binding State

In order to analyze the binding sites of CDK2 allosteric inhibitor and CDK2 ATP-competitive inhibitor on the same structure, 3PXF was also used to carry out docking study of the ATP binding site. However, the allosteric inhibitor ANS combined with 3PXF, which induced large conformational changes in the protein structure, especially in C-helix. Before carrying out the molecular docking of the ATP binding site of 3PXF, the conformation of 3PXF would be modulated by molecular dynamic. The ATP active binding site was defined by the residues, Lys33, Asp86, Leu83, Asn132, Lys89, and Asp145, which can affect the activity of the CDK2 ATP-competitive inhibitors [6].

Staurosporine, the CDK2 ATP-competitive inhibitor [6], was put into the defined binding site, and the protein-molecule complex was used as molecular dynamic starting point. The solvent environment was added to the complex system to simulate the actual situation by using Solvation protocol in DS. Then, the system was submitted to CHARMm by force-filing and Constraints were set as Harmonic Restraint. The system was relaxed by energy minimization, which was set at 10,000 steps of steepest descent and followed by 10,000 steps of conjugated gradient. The other parameters were set to defaults. Finally, the conformation of 3PXF with ATP-competitive inhibitor was obtained and the RMSD value between the modified comformation and the reported structure of CDK2-staurosporine was calculated to show the modified comformation was reliable.

##### Docking Strategy of the ATP Binding Site

The active ATP binding site was defined by the residues mentioned above based on the modulated protein structure. The CDK2 ATP-competitive inhibitors were docked into the active ATP site to verify the ability of the two docking algorithms mentioned in Section 3.3.1. Due to no initial compound like CDK2 ATP-competitive inhibitor binding with 3PXF, RMSD cannot used to evaluate the docking algorithm. The number of CDK2 ATP-competitive inhibitors which docked into the active ATP binding site was used to evaluate the docking algorithm. The more compounds docked into the binding site, the more reliable the docking algorithm. The reliable docking algorithm would be chosen to refine the list of potential CDK2 ATP-competitive inhibitors which was obtained by the HipHop model.

### 3.4. Cell Proliferation Assay

MTT assay was used to measure cell growth-inhibitory activity of the selected most promising compounds in HepG2 cell lines [26]. Cells were cultured in 96-well culture plate at 1 × 10^4^ cells/well. After 24 h cultured at 37 °C in the atmosphere of 5% CO_2_, cells were adhered and treated with different concentrations of the targeted compounds and incubated for 24 h. Then, the supernatants were discarded and MTT (0.5 mg/mL) was added to each well and incubated at 37 °C in 5% CO_2_ for an additional 4 h. Following, the MTT was removed and 150 μL of formazan in dimethylsulphoxide (DMSO) was added to terminate response and then plates were set to the table shaker for 5 min at low speed. Cell proliferation was evaluated by measuring the absorbance at 570 nm using ELISA Plate Reader (Molecular Devices, Los Angeles, CA, USA). The IC_50_ values were calculated by SPSS statistics 17.0.

## 4. Conclusions

Recently, owing to excellent selectivity and decreased side effects, pure CDK2 allosteric inhibitors discovery and design has gradually attracted a lot of attention. In this study, a new virtual screening strategy was provided and series of computer-aided drug design technologies were used to discover pure CDK2 allosteric inhibitors from TCM, and MTT assay was used to determine the proliferation effect of the promising allosteric compounds in HepG2 cell lines. To be specific, the GALAHAD pharmacophore model of CDK2 allosteric inhibitors and the HipHop pharmacophore model of CDK2 ATP-competitive inhibitors were constructed respectively. The optimal pharmacophore models, GALAHAD MODEL_007 and HipHop pharmacophore model Hypo1-1, were used to screen the TCMD. Then, the crystal structure of CDK2 (3PXF) with an allosteric pocket was used for molecular docking to refine the screening results of pharmacophore model and analyze the ligand-receptor interactions. In order to improve the reliability and accuracy of the screening result, the compound which interacted with the allosteric site and also interacted with the ATP site were discarded and the remaining compounds were regarded as the potential pure CDK2 allosteric inhibitors. Finally, prostaglandin E1 and NDGA were analyzed in detail and then the growth inhibitory effect of each was tested in HepG2 cell lines. CDK2 allosteric inhibitors have the effect of anti-cancer and the known CDK2 inhibitor, ANS, was used as a positive control. The results showed that prostaglandin E1 and NDGA exhibited anti-cancer effect in human HepG2 cell line with IC_50_ values in the μM range. It might suggest that the two promising CDK2 allosteric inhibitors interacted with CDK2 allosteric site to play the anti-cancer role. The results displayed—for the first time—that prostaglandin E1 and NDGA are promising CDK2 allosteric inhibitors, which also can be used as leading compounds for designing novel CDK2 allosteric inhibitors and anti-cancer drugs.

## Figures and Tables

**Table 1:** The validation results of each GALAHAD model for CDK2 allosteric inhibitors.

Model	Specificity	N_hits	Features	PARETO	Energy
MODEL_001	3.718	7	6	0	21.12
MODEL_002	4.049	7	5	0	1688.09
MODEL_003	4.638	7	7	0	1280.52
MODEL_004	2.085	7	8	0	11.17
MODEL_005	3.016	7	9	0	37.30
MODEL_006	4.587	7	8	0	11.08
MODEL_007	5.291	7	9	0	38.13
MODEL_008	5.093	7	6	0	21.67

**Table 2:** The validation results of the HipHop pharmacophore models for CDK2 ATP-competitive inhibitors.

Hypo	Features ^a^	Rank Score ^b^	TA ^c^	TD ^d^	Ha ^e^	Ht ^f^	*HRA* ^g^	*IEI* ^h^	*CAI* ^i^
1	RHAAEv5	133.141	23	92	21	40	91.30%	2.10	1.92
2	RHAAEv5	132.625	23	92	20	38	86.96%	2.11	1.83
3	RHAAEv5	132.431	23	92	21	39	91.30%	2.15	1.97
4	RHAAEv5	131.957	23	92	18	34	78.26%	2.12	1.66
5	RHDAEv5	131.796	23	92	22	43	95.65%	2.05	1.96
6	RHAAEv5	131.641	23	92	21	39	91.30%	2.15	1.97
7	RHAAEv5	131.180	23	92	19	35	82.6%	2.17	1.79
8	RHAAEv5	131.114	23	92	20	37	86.96%	2.16	1.88
9	RHAAEv5	130.381	23	92	20	37	86.96%	2.16	1.88
10	RHAAEv5	130.168	23	92	21	39	91.30%	2.15	1.97

^a^ H, hydrophobic; A, hydrogen bond acceptor; R, ring aromatic; D, hydrogen bond donor; Ev, Excluded Volumes; ^b^ The higher rank score indicated that the model might be well-mapped; ^c^ TA is the number of active compounds in the test set; ^d^ TD is the total number of compounds in the test set; ^e^ Ha is the hits number of active compounds searched by a pharmacophore model; ^f^ Ht is the total number of compounds searched by a pharmacophore model; ^g^ *HRA* indicates the ability to identify active compounds from the test set; ^h^ *IEI* indicates the ability to distinguish active compounds from inactive compounds; ^i^ *CAI* is the Comprehensive Appraisal Index.
